# Severe Inflammatory Croup

**Published:** 1856-04

**Authors:** 


### SEVERE INFLAMMATORY CROUP.

Mr. Martyn communicated to the Western Medical and Surgical Society of London, Nov. 16, 1855, an interesting case of severe inflammatory croup, in which alcoholic stimulants, freely administered, rescued the patient, a child aged four years, from imminent death. It had just recovered from the effects of scarlatina. The croupy symptoms became fully established in the course of twenty-four hours from first being observed. The fever was very high, and the obstruction to the circulation through the lungs very imminent, and the characteristic symptoms of croupy inspiration alarming. These symptoms were treated, as usual, with promptness and the ordinary remedies. The patient was put under the influence of tartar emetic and calomel, and a large blister was applied to the neck. The next day the symptoms continued as before, but towards evening, the third stage, or that of collapse, became established. The face became dusky, and the skin covered with a damp perspiration, the mouth and lips purple and congested, and the efforts at respiration alarming. Under these circumstances the antimonial and mercurial treatment was omitted, and recourse had to stimulants. He was ordered half an ounce of brandy, diluted, and a less quantity at intervals of an hour, or oftener, if it could be swallowed; this was accompanied by the administration of the chlorate of potassa every hour, in combination with good nourishment. The next day, though the breathing was very labored and difficult, the child was decidedly relieved. He continued to mend under the above treatment by brandy, though for some days the chest symptoms rendered his recovery doubtful. In the remarks which Mr. Martyn offered on the case, he considered that the time for the successful use of tartar emetic, &c., soon passed away, and that exhaustion soon waits upon the patient and demands the opposite kind of treatment. He considered that brandy was in every way preferable to any of the other stimulants used in the third stage of croup, as ammonia, senega, and the like; it had the advantage of being acceptable to children, and of producing more decided and permanent effects than any other stimulant. He was then led to make a few remarks upon the general lowering and evacuating treatment adopted in all acute diseases, the effect of which, in conjunction with the spare and starving diet, soon reduce the patient to such a state of exhaustion as to render him unable to combat the symptoms of disease. Should we not, then, recognize this element in all such diseases more readily than we do, and be ready at an early stage to administer alcoholic stimulants as soon as the evacuants employed have had fair play? Surely it is too late to delay their administration until exhaustion has too truly developed itself. Such stimulants do not augment inflammation, but maintian the vital powers whilst the natural sources of power, as nutrition, are cut off, and if stimulants do not directly combat inflammation, they surely, by supporting the system, give opportunity and fresh impulse to those vital forces that are always tending to subdue inflammations.

